# Local Wireless Sensor Networks Positioning Reliability Under Sensor Failure

**DOI:** 10.3390/s20051426

**Published:** 2020-03-05

**Authors:** Javier Díez-González, Rubén Álvarez, Natalia Prieto-Fernández, Hilde Perez

**Affiliations:** 1 Department of Mechanical, IT and Aerospace Engineering, Universidad de León, 24071 León, Spain; nprief02@estudiantes.unileon.es (N.P.-F.); hilde.perez@unileon.es (H.P.); 2 Positioning Department, Drotium, Universidad de León, 24071 León, Spain

**Keywords:** cramer rao lower bound, localization, LPS, multi-objective optimization, sensor failure, wireless sensor networks

## Abstract

Local Positioning Systems are collecting high research interest over the last few years. Its accurate application in high-demanded difficult scenarios has revealed its stability and robustness for autonomous navigation. In this paper, we develop a new sensor deployment methodology to guarantee the system availability in case of a sensor failure of a five-node Time Difference of Arrival (TDOA) localization method. We solve the ambiguity of two possible solutions in the four-sensor TDOA problem in each combination of four nodes of the system by maximizing the distance between the two possible solutions in every target possible location. In addition, we perform a Genetic Algorithm Optimization in order to find an optimized node location with a trade-off between the system behavior under failure and its normal operating condition by means of the Cramer Rao Lower Bound derivation in each possible target location. Results show that the optimization considering sensor failure enhances the average values of the convergence region size and the location accuracy by 31% and 22%, respectively, in case of some malfunction sensors regarding to the non-failure optimization, only suffering a reduction in accuracy of less than 5% under normal operating conditions.

## 1. Introduction

Autonomous navigation has meant a challenge for scientific development over the last few years. The high accuracy required has entailed the interest in Local Positioning Systems (LPS) where the positioning signal paths are reduced between targets and architecture sensors. This fact significantly reduces noise and uncertainties by minimizing the global architecture errors with respect to Global Navigation Satellite Systems (GNSS). 

GNSS provide global coverage but the distortion of their signals in their travel affects the stability and the accuracy of the localization over time. In addition, GNSS navigation is denied in indoor environments, where Automatic Ground Vehicles (AGVs) mostly operate, as signals deteriorate crossing large buildings. This causes Non-Line-of-Sight (NLOS) connections between satellites and targets which makes position determination impractical. The application of also GNSS has limitations in outdoor environments such as low-altitude flights in Unmanned Aerial Vehicles (UAVs) due to the higher uncertainty in the vertical coordinate of the global systems. It is a consequence of the similar altitude of the satellites in their constellations.

These reasons have promoted this new localization concept based on LPS especially for high accuracy automated navigation [1,2]. LPS require the deployment of architecture sensors in a defined and known space where the capabilities of the system are maximized. The characteristics of the LPS for a defined space rely on the measurement of the physical magnitude used for the determination of the target location: time [3], power [4], frequency [5], angle [6], phase [7] or combinations of them [8].

Among these systems, the most extended are time-based models due to their reliability, stability, robustness and easy-to-implement hardware architectures. Time-based positioning has two main systems that differ in time measurements computed: Time of Arrival (TOA) [9] and Time Difference of Arrival (TDOA) [10] systems.

TOA systems measure the total time of flight of a positioning signal from an emitter to a receiver. It requires the synchronization of the clocks of all the system elements (i.e. targets and sensors). This leads to the generation of a sphere of possible locations in the 3-D space for each received signal in a different architecture sensor. The intersection of spheres determines the target location. Mathematical standards show that the unequivocal target location is achieved in TOA systems with at least four sensors.

TDOA systems compute the relative time between the reception of the positioning signal in two different architecture sensors. The synchronization of these systems is optional considering asynchronous TDOA architectures in which the time differences are computed in a single clock of a coordinator sensor [11] and synchronous TDOA where all architecture sensors must be synchronized. Time relative measurements lead to hyperboloid surfaces of possible location of targets. A hyperboloid equation is obtained every two architecture sensors while only (*n-1*) independent equations can be processed from *n* different sensors [12]. The required number of sensors to determine unequivocally the target location is five sensors for 3-D positioning in these methodologies. 

However, the intersection of three different spheres -3 architecture sensors- in TOA systems and three different hyperboloids -4 architecture sensors- in TDOA systems leads to two different potential solutions. Nevertheless, these solutions are not able to be discarded from a mathematical point of view.

In one of our previous works [13], we have demonstrated that a reliable unique solution to the intersection of three hyperboloids or spheres can be obtained through the maximization of the distance between the two potential solutions in a defined environment by means of Genetic Algorithms (GA). We achieve this result by applying Taylor-based algorithms [14] from an initial iteration point which must be close enough to the final solution. Results show that the node deployment has a direct impact in this finding.

The sensor distribution also has relation with the global accuracy of the LPS. Traditionally, the Position Dilution of Precision (PDOP) has been used to determine the achievable accuracy of time-based positioning systems in GNSS [15] by considering satellite location with respect to target nodes. This methodology considers the homoscedasticity of the satellite signals as they actually travel similar distances from satellites to target nodes. This consideration is impractical for LPS since the paths traveled can significantly differ from one architecture sensor to another producing the heteroscedasticity in the time measurements [16]. 

This fact promotes the use of Cramer Rao Lower Bound (CRLB) [17,18] derivations to characterize the White Gaussian Noise (WGN) present in the time measurements. In practice, CRLB determines the minimum achievable error in positioning systems [19]. We have computed these derivations for asynchronous and synchronous TDOA positioning methodologies in our recent works [20,21] in order to define the beauty of a node deployment in terms of accuracy. This has allowed us to perform the node deployment optimization in TDOA systems by means of GA. The reason of the use of heuristic techniques relies on the NP-Hard problem solution of the 3D sensor deployment in LPS and it is widely considered in the literature [22,23,24,25,26,27].

However, any of the approaches presented considers a possible sensor failure during the node distribution optimization addressed. This means that in these sensor deployments a sensor fault will cause the unavailability of TOA architectures with 4 sensors and TDOA architectures with 5 sensors. However, our finding in [13] has determined that an unequivocal solution for these systems with a possible sensor failure -3 sensors in TOA and 4 sensors in TDOA- can be achieved under an optimized node localization. As a consequence, an optimized sensor distribution can guarantee the availability of the system in sensor failure conditions through the consideration of a methodology to enhance the system properties in these situations.

**Figure 1:**
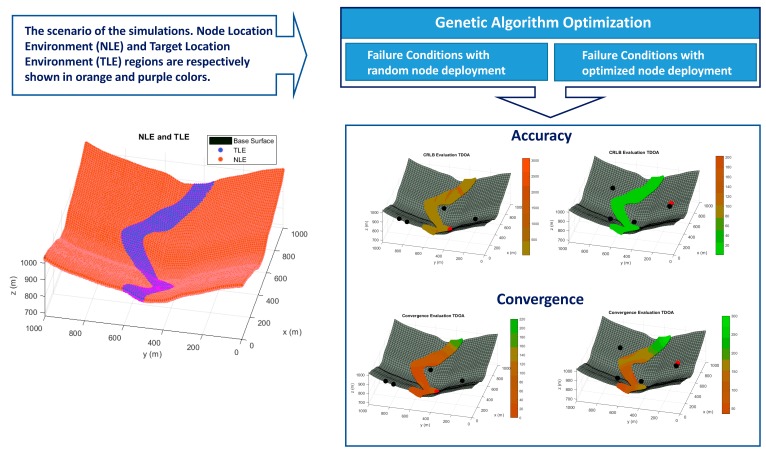
Graphical Abstract.

In this paper, we propose for the first time a GA optimization for the 3D node deployment in a TDOA system with five architecture sensors with failure consideration, maximizing the performance during regular operation and in any possible sensor malfunction (see Figure 1). For that purpose, we performed a multi-objective optimization in which we looked for a trade-off between the global accuracy of the system with five sensors and every combination of four nodes in a defined environment of an LPS. Additionally, we ensured the unequivocal position determination for every distribution of four sensors by maximizing the distance between the two possible mathematical solutions of the target location [11]. Based on [19] a 3D sensor distribution in irregular environments is provided, enabling the application of this failure-consideration approach to outdoor and indoor scenarios. This methodology will also ensure the availability of the system with acceptable accuracy in case of a sensor failure in any of the architecture nodes. 

The remainder of the paper is organized as follows: the algorithm for the target unequivocal location determination is presented in Section 2, the CRLB modeling is introduced in Section 3, the GA and the fitness function are presented in Section 4 and Section 5 and Section 6 show the results and conclusions of the presented paper.

## 2. Taylor-Based Positioning Algorithm in Time Difference of Arrival (TDOA) Systems

Relative time measurements in TDOA systems lead to hyperboloid equations of possible target locations. These equations are non-linear so numerical methods are required to solve the intersection of the hyperboloids. The algorithms used have been classified in two main categories: closed-form algorithms and iterative methods.

Closed-form algorithms [28,29] provide a direct final solution by solving a linearization of the hyperboloid equations. Iterative methods perform a gradient descent to achieve the solution through Taylor-Based linearization. These methods start from an initial position which must be closed enough to the final solution [30] to iteratively converge to the target location. The convergence of the algorithm depends on the initial position -usually the last known position of the target- which promotes a constant updating of the target location. 

The position calculation with four architecture sensors in TDOA systems provides two possible ambiguous target localizations. The achievement of an unequivocal position cannot be determined according to mathematical standards. As a consequence, the position determination by means of iterative methods provides a unique solution that it might not match with the real target location. Nevertheless, we have shown in [13] that the optimal solution can be achieved by maximizing the radius of convergence of the initial iteration point which forces the iterative method to converge to the real solution in a high confidence interval. It has been demonstrated that this fact coincides with the maximization of the distance between the two possible solutions in LPS. This allows us to solve the 3-D TDOA problem with 4 nodes through Taylor-Based positioning algorithms with enough confidence under the optimization proposed.

This finding enables LPS architectures of 5 sensors -minimum number of sensors to supply unequivocal target location- to provide stable and accurate service in case of sensor failure or temporal unavailability of one of the architecture nodes.

Taylor-Based algorithms in TDOA systems are linearizations of the equation of the time difference of arrival:

(1) $$\begin{array}{ll} R_{ij}=d_{ij}=d_{Ei} & -d_{Ej}=c\;t_{ij}=c\;\left(t_{i}-t_{j}\right)\\ & =\sqrt{{\left(x_{E}-x_{i}\right)}^{2}+{\left(y_{E}-y_{i}\right)}^{2}+{\left(z_{E}-z_{i}\right)}^{2}}\\ & -\sqrt{{\left(x_{E}-x_{j}\right)}^{2}+{\left(y_{E}-y_{j}\right)}^{2}+{\left(z_{E}-z_{j}\right)}^{2}} \end{array}$$

where $R_{ij}$ and $d_{ij}$ represent the distance difference of the signal travel from the emitter to sensors i and j, $d_{Ei}$ and $d_{Ej}$ are total distance from the emitter (*E*) to sensors *i* and *j*, *c* is the speed of the radioelectric waves, $t_{ij}$ is the time difference of arrival measured in the architecture sensors,$\;t_{i}$ and $t_{j}$ is the total time of flight of the positioning signal from emitter to receivers i and j respectively and $\left(x_{E}\;,\;y_{E}\;,z_{E}\right)$, $\left(x_{i}\;,\;y_{i}\;,\;z_{i}\right)$ and $\left(x_{j\;},\;y_{j}\;,\;z_{j}\right)$ are the Cartesian coordinates of the target and the sensors i and j.

Taylor approximation truncated on first order is applied in Equation (1) to linearize the equation from an initial iteration point $\left(x_{0}\;,\;y_{0}\;,\;z_{0}\right)$:

(2) $$R_{ij}=ct_{ij}=R_{ij_{0}}+\frac{\partial R_{ij}}{\partial x}∆x+\frac{\partial R_{ij}}{\partial y}∆y+\frac{\partial R_{ij}}{\partial z}∆z$$

where $R_{ij_{0}}$ is the range difference of arrival in the initial iteration point, $\frac{\partial R_{ij}}{\partial x},\;\frac{\partial R_{ij}}{\partial y}$ and $\frac{\partial R_{ij}}{\partial z}$ are the partial derivatives of the range differences measured in the *i* and *j* architecture sensors particularized in the initial iteration point.

The application of this process to sensors k and l to complete the four-sensor 3D TDOA problem solution in [13] generates the range difference matrix (∆R):

(3) $$∆R=\left(\begin{array}{ccc} \begin{array}{c} \frac{\partial R_{ij}}{\partial x}\\ \frac{\partial R_{il}}{\partial x}\\ \frac{\partial R_{ik}}{\partial x} \end{array} & \begin{array}{c} \frac{\partial R_{ij}}{\partial y}\\ \frac{\partial R_{il}}{\partial y}\\ \frac{\partial R_{ik}}{\partial y} \end{array} & \begin{array}{c} \frac{\partial R_{ij}}{\partial z}\\ \frac{\partial R_{il}}{\partial z}\\ \frac{\partial R_{ik}}{\partial z} \end{array} \end{array}\right)\left(\begin{array}{c} ∆x\\ ∆y\\ ∆z \end{array}\right)=H∆P$$

where H is the partial derivative matrix, usually known as the visibility matrix, and ∆P represents the coordinate variances in each space direction which is the unknown of the equation. The previous equation is solved and iterated until no changes in coordinate variances are appreciated by means of the least squares method as follows:

(4) $$∆P={\left(H^{t}H\right)}^{-1}H^{t}∆R=\left(\begin{array}{c} ∆x\\ ∆y\\ ∆z \end{array}\right)\;$$

## 3. Cramer Rao Lower Bound (CRLB) Modeling in TDOA Systems

CRLB is an unbiased estimator of the lowest variance of a parameter. Its usage in the localization field is widespread [31,32,33] since it allows us to determine the minimum achievable error by the system analyzed. 

It characterizes the WGN present in the time measurements of the time-based positioning systems. The uncertainties introduced in the measurements depend on the distance traveled by the positioning signal from the emitter to the architecture sensors in a heteroscedastic noise consideration. Recent studies [18] developed a matrix form of the CRLB considering heteroscedasticity in time measurements:

(5) $$\begin{array}{ll} FIM_{mn}={\left(\frac{\partial h\left(TS\right)}{\partial TS_{m}}\right)}^{T} & R^{-1}\left(TS\right)\left(\frac{\partial h\left(TS\right)}{\partial TS_{n}}\right)\\ & +\frac{1}{2}tr\left\{R^{-1}\left(TS\right)\left(\frac{\partial R\left(TS\right)}{\partial \mathrm{TS}}\right)R^{-1}\left(TS\right)\left(\frac{\partial R\left(TS\right)}{\partial TS_{n}}\right)\right\} \end{array}$$

where FIM indicates the Fisher Information Matrix, m and n are the sub-indexes of the estimated parameters in FIM, TS is the target sensor Cartesian coordinates, h(TS) is a vector that contains the travel of the signal in the TDOA architecture to compute a time measurement:

(6) $$\begin{array}{c} h_{TDOA_{i}}=\|TS-CS_{i}\|-\|TS-CS_{j}\|\\ i=1,\;\ldots ,\;N_{CS}\\ j=1,\;\ldots ,\;N_{CS} \end{array}$$

being $CS_{i}$ and $CS_{j}$ the coordinates of the architecture sensors *i* and *j* and $N_{CS}$ the number of sensors involved in the position determination. R(TS) is the covariance matrix of the time measurements in the architecture sensors. The covariance matrix is built with a heteroscedastic noise consideration in the sensors modeled by a Log-normal path loss propagation model [21] obtaining the following variances:

(7) σTDOAij2=c2B2(PTPn)PL(d0)[(dEid0)n+(dEjd0)n]i=1, …, NCS  j=1, …, NCS where i≠j

where B is the signal bandwidth, $P_{T}$ is the transmission power, $P_{n}$ is the mean noise level determined through the Johnson-Nyquist equation, n is the path loss exponent, $d_{0}$ is the reference distance from which the path loss propagation model is applied and $PL\left(d_{0}\right)$ is the path-loss in the reference distance.

The inverse of the Fisher Information Matrix (J) provides in its diagonal the uncertainties associated with each variable to estimate, i.e. the three Cartesian coordinates of the target for a 3D positioning. The location accuracy is directly evaluated through the Root Mean Squared Error (RMSE), which is computed based on the trace of the J matrix.

(8) $$RMSE=\;\sqrt{J_{11}+J_{22}+J_{33}}=\sqrt{\sigma_{x}{}^{2}+\sigma_{y}{}^{2}+\sigma_{z}{}^{2}}$$

This model will be applied in the GA optimization with five sensors and each distribution of four sensors in any possible target location in the defined scenario in order to compare the beauty of different node deployments.

## 4. Genetic Algorithm (GA) Optimization

The strong influence of the sensor placement in the LPS performance enables the maximization of their capabilities through the optimization of their sensor distribution. This approach is especially suitable in complex 3D environments, where the most important source of positioning error is promoted by the sensor distribution. 

In this work, we developed an optimization methodology to locate the positioning sensors of a five-sensor TDOA system with the consideration of an eventual failure in some of the system nodes. This procedure must guarantee the convergence of the iterative algorithm with all the possible combinations of four nodes in every target location under coverage. Furthermore, the achievement of an optimized node distribution for the normal operating conditions with five system nodes must be accomplished. This leads to a multi-objective optimization which considers both normal and failure operating conditions.

In our previous works [21], a GA for optimizing sensor distributions in 3D irregular environments is presented. The proposed methodology allows a free definition of the optimization region and the reference surface for locating the sensors of the positioning architecture. In addition, the procedure is modular, allowing the election of different selection techniques, percentage of elitism, crossover methodologies, mutation types, and convergence criteria. 

After the choice of the optimization method, the next step is the definition of the fitness function. In this case, a multi-objective optimization is carried out for maximizing the accuracy of the TDOA architecture when the minimum number of sensors for positioning is available, i.e. when some of the architecture sensors fail. Accordingly, the methodology proposed in [13] guarantees the attainment of a unique location in TDOA architectures with 4 sensors by the Taylor-based positioning algorithm described in Section 2, based on an initial iteration point closed to the target estimation. The region where this procedure converges to the final solution depends on the geometric properties of the target and the architecture sensors, i.e. the sensor placement in the environment. Based on this relation, the regions of convergence can be maximized through the optimization of the sensor distribution [13].

Consequently, the goal of the multi-objective optimization is the combined maximization of the TDOA system accuracy in 3D environments when the whole architecture is available and when only four sensors are accessible, limited by the size of the convergence regions that allow the correct execution of the Taylor-based positioning algorithm. The fulfillment of these objectives guarantees the robustness of the TDOA architectures in adverse conditions of operation. The fitness function is detailed hereafter:

(9) $$\begin{array}{ll} ff=\overset{Comb}{\underset{1}{\sum }}\{\frac{C_{1}}{NT}\sum & \left\{1-\frac{{\left[\left(\frac{1}{RMSE_{ref}}\right)-\left(\frac{1}{RMSE_{4sensors}}\right)\right]}^{2}}{{\left(\frac{1}{RMSE_{ref}}\right)}^{2}}\right\}\\ & +\frac{C_{2}}{NT}\sum \left\{\frac{{\left[\left(\frac{1}{D_{ref}}\right)-\left(\frac{1}{D}\right)\right]}^{2}}{{\left(\frac{1}{D_{ref}}\right)}^{2}}\right\}\}\\ & +C_{3}\sum \frac{\left\{1-\frac{{\left[\left(\frac{1}{RMSE_{ref}}\right)-\left(\frac{1}{RMSE_{Ncs}}\right)\right]}^{2}}{{\left(\frac{1}{RMSE_{ref}}\right)}^{2}}\right\}}{NT}-C_{4}\frac{\sum_{i=1}^{N_{CS}}BL_{i}}{N_{CS}} \end{array}$$

where Comb is the number of groups of four sensors which are obtainable based on the total number of architecture sensors, *NT* is the number of analyzed points, $RMSE_{ref}$ is the reference accuracy, $RMSE_{4sensors}$ is the vector that contains the CRLB evaluation for each point at analysis with each combination of 4 sensors, $D_{ref}$ indicates the reference distance for the convergence criteria, D represents the vector that specifies the convergence evaluation in terms of the distance between the two possible solutions (combinations of 4 sensors) for each point at study, $RMSE_{Ncs}$ is the vector that contains the CRLB analysis for each point at study when all architecture sensors are available, $C_{1}$,$\;C_{2}$, $C_{3}$ and $C_{4}$, are coefficients for calibration of the individual summands of the fitness function, and $BL_{i}$ is the penalization factor associated with the existence of sensors in banned regions (if they exist).

The implemented fitness function presents two important characteristics: the individual summands of the function are confined in the interval (0,1], enabling different ponderations for the optimization; and the $RMSE_{ref}$ and $D_{ref}$ magnitudes are adaptive to the problem characteristics, facilitating the diversification and intensification phases of the GA in complex environments.

## 5. Results

In this section, the results of the optimization for sensor failure in TDOA architectures are presented. Initially, a 3D complex scenario was designed for carrying out the optimization, proving the adaptability of the proposed methodology in any environment. For this purpose, an irregular scenario of simulation was designed by considering any possible target location and extensive available zones for positioning the architecture sensors in the environment of simulations. This fact ensures the versatility of the procedure for its application to indoor and outdoor environments.

**Figure 2:**
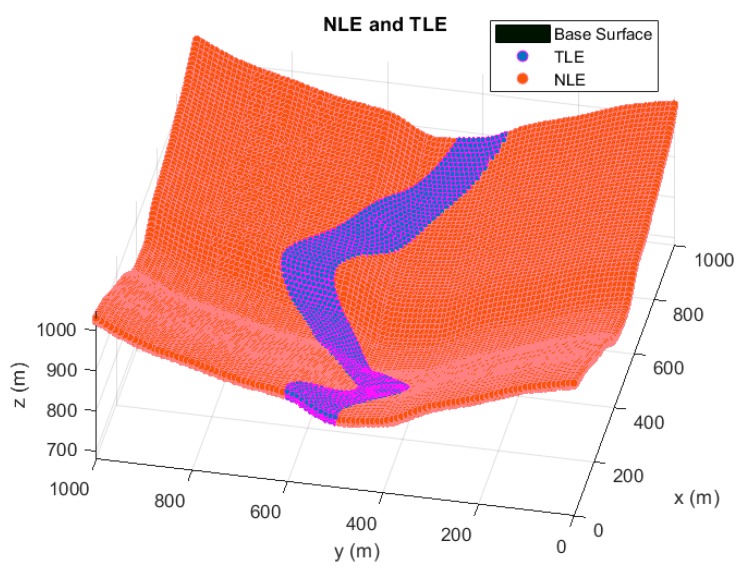
The scenario of simulations. The reference surface is depicted is grey tones. Node Location Environment (NLE) and Target Location Environment (TLE) regions are respectively shown in orange and purple colors. The discretized points of the TLE zone are the points employed for the optimization of the Time Difference of Arrival (TDOA) architecture performance. In the case of the NLE area, the points shown are only a representation of the area where every sensor can be located.

In Figure 2, the term TLE represents the Target Location Environment which defines the region where targets are possible to be located. For this simulation, the TLE region extends from 0.5 to 15 m of elevation from the base surface, emulating the operating conditions for a positioning system in the proximity of the ground. TLE region is spatially discretized based on a division of 20 m in x and y coordinates, and 2 m in z coordinate. This ensures the correct evaluation and continuity of the accuracy and convergence analysis, and the restriction in the total number of the studied points.

The NLE area expresses the Node Location Environment, which indicates all possible sensor locations. In the case of the NLE region, the height of the sensors is limited in the range of 3 to 10 m from the base surface, depicting for a typical outdoor LPS implementation. The discretization of the NLE region depends on the codification of the individuals of the GA, precisely on the longitude of the chromosomes implemented. In this way, the resolution of the NLE area varies in the three Cartesian coordinates from 0.5 to 1 m, alluring a fine setting in the optimization of each sensor.

Table 1 and Table 2 show the principal parameters of configuration for the positioning system and the GA characteristics applied for the optimization.

Values presented in Table 1 were chosen in an attempt to stand for a generic positioning technology, expressed by the typical parameters of transmission power, frequency of emission and bandwidth. The configuration of the GA shown in Table 2 has been the subject of deep analysis, looking for the trade-off between the fitness function maximization and convergence speed.

**Figure 3:**
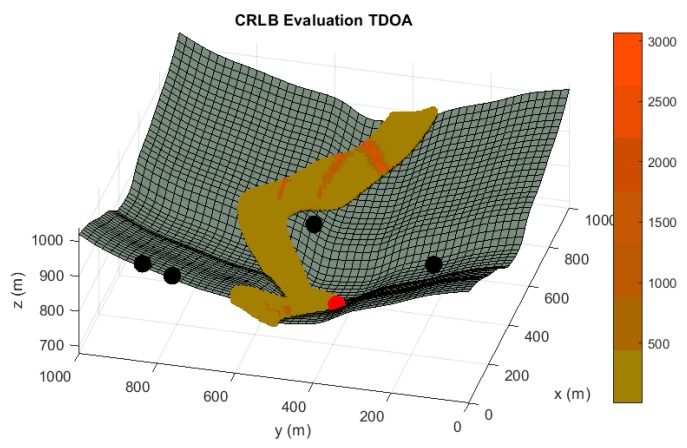
Accuracy analysis in terms of Cramer Rao Lower Bound (CRLB) in meters for a random sensor distribution of five sensors, under the assumption of one randomly malfunction sensor. Black spheres indicate the location of active sensors and red spheres highlights the sensor which is not available. Red tones in the color bar indicate bad accuracy evaluations, while green tones imply acceptable accuracy values.

**Figure 4:**
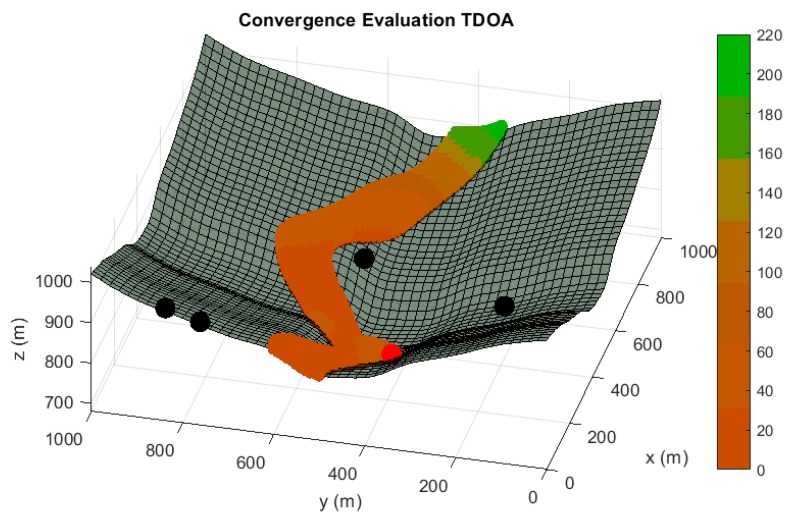
Convergence radius analysis in meters for a random sensor distribution of 5 sensors, under the assumption of one randomly malfunction sensor. The convergence radius represents the maximum radius of the sphere of convergence in which every inside point used as initial iterating point of the positioning algorithm guarantees the unequivocal position determination by using the four available sensors. It represents the same operating condition than Figure 2. Red tones in the color bar indicate bad convergence radius values, while green tones imply acceptable convergence magnitudes.

In the following paragraphs and figures, the results after the optimization process are shown for distributions of 5 sensors. Firstly, in order to highlight the importance of the sensor distribution, a random sensor placement is evaluated in terms of accuracy and convergence under a sensor failure in Figure 3 and Figure 4. 

**Figure 5:**
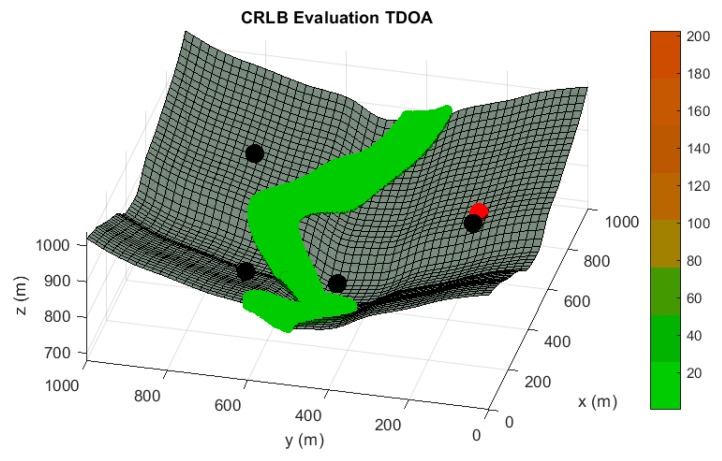
Accuracy analysis in terms of CRLB in meters for the optimized distribution of 5 sensors under possible failure. The condition represented corresponds with the Case I - Sensor Fail 1 of Table 3. Red tones in the color bar indicate badly accuracy evaluations, while green tones imply acceptable accuracy values.

**Figure 6:**
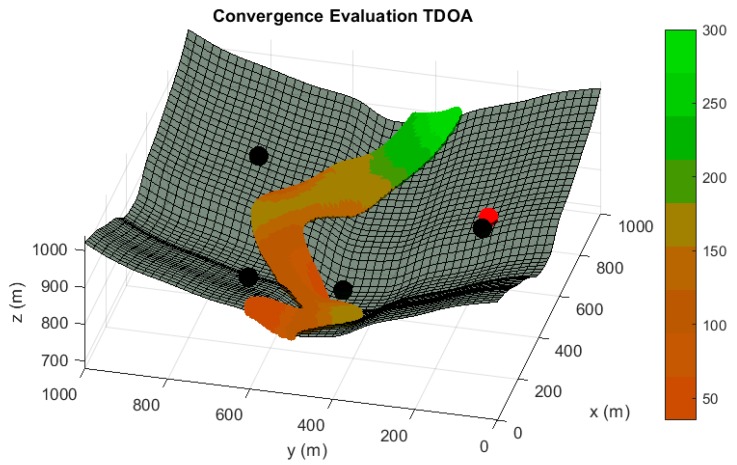
Convergence radius analysis in meters for the optimized distribution of 5 sensors under possible failure. The condition represented corresponds with the Case I - Sensor Fail 5 of Table 3. Red tones in the color bar indicate badly convergence radius values, while green tones imply acceptable convergence magnitudes.

As it is shown, the performance of this sensor distribution is not acceptable for any positioning service. The results for the optimized sensor placement with failure consideration, 5 sensors nominal operating conditions and convergence maximization (Case I) are provided in Figure 5 and Figure 6 when one of the sensors is not available.

The benefits of the consideration of the sensor failure in the architecture design have been shown through the differences in accuracy and convergence from the Figure 2, Figure 3, Figure 4 and Figure 5. However, a comparison of the performance of the methodology proposed in this paper with a conventional optimized node distribution in which the failure conditions are not considered is needed to conclude the beauty of the technique. In Table 3, we set the parameters considered in each optimization considering nominal operation, failure conditions and convergence (Case I) and only nominal operating conditions (Case II). Case II match up with the GA optimization that we previously proposed in [21].

In Table 4, a comparison between the optimized sensor distribution for sensor failure (Case I) and the optimized sensor placement of 5 sensors without malfunction consideration and convergence maximization (Case II) is supplied. It should be stressed that this last optimization is carried out through a fitness function with the direct evaluation of the CRLB for 5 sensors and the last term of the Equation (8).

Table 4 and Table 5 show the importance of the optimization of the sensor distribution under possible sensor failure. This feature is especially remarkable in the analysis of the convergence radius when some of the sensors are not available for positioning. 

The results of these tables reveal that the optimization carried out in Case I not only minimizes the CRLB (i.e. maximum achievable accuracy based on the conditions of operation) when only 4 sensors are accessible, it also maximizes the region where the Taylor-based positioning algorithm is able to operate (together with alliterative methods).

Optimizations with failure-consideration (Case I) increase the radius of convergence by 30.9 % in failure conditions while they also experience a boost of 8.5% in this confidence interval in the normal operating condition of five sensors availability. This is due to the convergence radius maximization in the failure-consideration optimization which is not considered in conventional sensor deployment methodologies. This shows that an increase in this confidence interval in the distributions of four sensors has also a direct effect in the convergence radius of the five-sensor normal operating distribution of the failure-consideration optimization. 

The beauty of this combined multi-objective optimization is that the accuracy of the four-sensor combinations in failure conditions has been increased by 22% while the accuracy of the normal operating five sensor distribution (Case I) has been reduced by less than 5% with regards to conventional node deployments (Case II) that only consider the five-sensor optimization. 

Furthermore, the achievement of higher values of the convergence radius in the failure-consideration optimization enhances availability and security in failure conditions by solving the ambiguity of two valid mathematical solutions and by increasing the confidence interval of applying Taylor-Based positioning algorithms in normal operating conditions with regards to conventional node deployment methodologies. 

This new optimization procedure considering sensor failures does guarantee the robustness of the positioning system in complex conditions of operations, and the design of architectures considering these situations.

## 6. Discussion

The location of sensors in LPS has been an active topic of research over the last few years [3,13,21,22,23,24]. This is a consequence of its direct relation with the accuracy, stability and robustness of wireless local sensor networks. Conventional approaches to the optimal node distributions have considered the best location of the sensors for nominal operating conditions.

Nevertheless, in actual implementations of the LPS, some sensors are possibly denied for positioning due to the presence of obstacles that disturb signals introducing adverse effects such as multipath or signal deterioration. Furthermore, a possible sensor malfunctioning introducing noise in the measurements must be considered. 

These facts have not been studied in previous sensor distribution optimizations. In this work, we propose for the first time in the authors’ best knowledge a node deployment methodology that enhances position determination in case of a sensor failure. Additionally, we apply this process to the more restrictive TDOA system to unequivocally determine target location, i.e. five-sensor TDOA deployments. This leads to a sensor-failure configuration in which we first need to solve the position ambiguity determination in systems with only four nodes according to the finding that we proposed in [11].

For this purpose, we performed a multi-objective optimization in a defined 3D irregular scenario in order to extrapolate the results to normal LPS applications. This optimization reduces the CRLB while it is also maximizing the radius of convergence of the Taylor-Based algorithm that we use for the target location determination. 

Results show the beauty and importance of this new technique as it is able to enhance the system behavior in failure conditions with regards to only nominal optimizations. This is particularly remarkable since conventional optimization approaches are only focused in nominal operating conditions of LPS and they can suffer from temporal unavailability that can motivate important drawbacks in autonomous navigation.

## 7. Conclusions

Local Positioning Systems have emerged over the last few years for high-demanded accurate applications. Among them, time-based positioning architectures become predominant for its robustness, stability and trade-off between accuracy and complexity.

In this paper, we propose a method to guarantee system availability under sensor failure. This is a key factor for the real operation of LPS as a consequence of the possible ineffective link between target and sensors in complex environments and possible sensor malfunctioning.

In order to simulate an actual operation environment, we have defined a 3D irregular scenario consisting of a five-sensor deployment of a TDOA architecture. This configuration validates the methodology proposed for terrestrial and aerial applications in indoor and outdoor environments. In TDOA architectures, an unequivocal target location can be determined with a minimum of five sensors according to mathematical standards. However, we have proved that the ambiguity in the position determination with four sensors can be solved by the used of Taylor-Based positioning algorithms in a convergence region around the true target location which, in practice, corresponds with the maximization of the two possible solutions distance. 

The achievement of this disambiguation can be obtained through an optimized sensor distribution. The node deployment must also minimize the time measurement uncertainties which are characterized by means of the CRLB. For this reason, we implement a multi-objective optimization for the combined maximization of the accuracy and convergence under each possible sensor failure condition. In addition, the optimization needs to guarantee the reduction of the uncertainties for the nominal performance with five sensors.

Results show that the proposed method can attain both accuracy and convergence requirements under every possible sensor failure condition. The global optimization with five sensors without sensor failure consideration overcomes the five-sensor deployment optimization with failure consideration in terms of medium accuracy during nominal operation by less than 5%. In contrast, in circumstances where some of the sensors are not available and only 4 sensors can be applied in the target position calculation, the optimization considering sensor failure increases the average values of convergence region size and accuracy by 30.9% and 22% respectively, regarding the non-failure optimization. These results show the importance of considering the anomaly cases of sensor failure during the LPS node distribution optimization in order to guarantee availability and operation quality in high-demanding accuracy applications.

## Figures and Tables

**Table 1:** Parameters of configuration for the positioning system operation. Their selection is based on [19,34].

Parameter	Value
Transmission power	100 W
Mean noise power	−94 dBm
Frequency of emission	1090 MHz
Bandwidth	100 MHz
Path loss exponent	2.05
Antennae gains	Unity
Time-Frequency product	1

**Table 2:** Configuration of the principal elements of the Genetic Algorithm (GA).

GA	Selection
Population size	90
Selection technique	Tournament 2
% Elitism	5
Crossover technique	Single-point
% Mutation	3
Convergence criteria	80% individuals equals

**Table 3:** Definition of the parameters considered for optimization in Case I and Case II.

Parameter Considered	Case I	Case II
Nominal Operating Conditions (5 sensors distribution)	✓	✓
Failure Conditions (4 sensors distributions)	✓	**X**
Convergence Maximization	✓	**X**

**Table 4:** Comparative between the optimizations of Case I and II.

Sensor Distributions	Sensor Fail	CRLB Evaluation TDOA (meters)			Convergence Evaluation (meters)		
		Max	Mean	Min	Max	Mean	Min
Case I	Sensor 1	62.408	0.651	0.233	300	138.684	35
	Sensor 2	133.556	0.875	0.216	240	125.786	40
	Sensor 3	117.304	0.627	0.223	280	154.237	40
	Sensor 4	191.480	2.005	0.196	300	138.851	35
	Sensor 5	188.676	7.425	0.237	220	129.149	4
	None	0.795	0.326	0.154	300	140.229	40
Case II	Sensor 1	206.049	1.340	0.225	240	103.711	2
	Sensor 2	159.772	1.512	0.149	280	84.650	2
	Sensor 3	65.487	1.688	0.169	220	102.037	4
	Sensor 4	199.168	0.629	0.182	260	113.604	2
	Sensor 5	2340.42	9.674	0.181	240	70.850	2
	None	0.872	0.312	0.143	300	128.306	10

**Table 5:** Comparative between the optimizations of Case I and II. Values presented show the comparison in relative terms of the failure consideration distribution regarding the optimization for normal operation of the system.

Performance Analysis		Case I	Case II	Sensor Distribution: Case I vs Case II
Mean CRLB Evaluation TDOA (meters)	Failure conditions	2.316	2.969	−22.0 %
	Non-Failure conditions	0.326	0.312	+4.3 %
Mean Convergence Evaluation (meters)	Failure conditions	137.341	94.970	+30.9 %
	Non-Failure conditions	140.229	128.306	+8.5 %

## Abbreviations

The following abbreviations are used in this manuscript:

AGVs	Automatic Ground Vehicles
CRLB	Cramer Rao Lower Bound
FIM	Fisher Information Matrix
GA	Genetic Algorithm
GNSS	Global Navigation Satellite Systems
LPS	Local Positioning Systems
NLOS	Non-Line-of-Sight
PDOP	Position Dilution of Precision
RMSE	Root Mean Square Error
TDOA	Time Difference of Arrival
TOA	Time of Arrival
TS	Target Sensor
UAVs	Unmanned Aerial Vehicles
WGN	White Gaussian Noise
